# The Non-Panel Movement: Dr. Edwin Smith on Its Position and Prospects

**Published:** 1914-03-28

**Authors:** 


### THE NON-PANEL MOVEMENT.

### Dr. Edwin Smith on its Position and Prospects.

The weekly pages of The Hospital devoted to official details of the transactions and doings of the National Medical Union have familiarised our readers, whether medical men or institutional officers, with the active work that it is daily carrying on. But the interest which this weekly official chronicle has created, hardly less outside the Union's members than within, has led us this week to seek a comprehensive view of the Non-Panel Movement as a whole, for which purpose our Commissioner visited Dr. Edwin Smith, the chairman of the Wandsworth Non-Panel Association—from the first one of the most active centres of opposition to the Insurance Act—who courteously gave his estimate of the position and prospects of non-panel practitioners at the present time.

### Is the Amendment of the Act Possible?

Asked how far the National Medical Union had succeeded in co-ordinating into a defined policy the views of the different Non-Panel Associations which it was formed to organise, Dr. Edwin Smith said: "The present committee of the National Medical Union had a very difficult task entrusted to it, but I understand that the work of co-ordinating the Non-Panel Associations of the country has been most successfully carried out, and that a meeting has just been held to consider a number of suggestions as to policy sent up at the invitation of the Union by the various federated societies."

"Have the suggestions from the local Associations been difficult to reconcile?"

"This is a question for the officials of the Union, but it may safely be answered in the negative. I believe that differences have been strikingly few, and that where they have existed they have had reference to minor points. All non-panel men are unanimous in their opposition to the medical arrangements under the National Insurance Act, but views may vary in matters of detail. There are those who do not believe that the present Act can be satisfactorily amended, while others believe that attempts should be made to modify it. My personal view has always been that the amendment of the Act is impracticable, because any amendment to meet the rightful demands of non-panel practitioners would have to be so drastic that in practice such amendment would mean re-drafting the medical benefit sections of the Act."

### "A Flat Rate is Medically Impossible."

"Has the policy of aloofness so far justified itself?"

"I think undoubtedly that it has, but, before pointing out to you why, it is clear that no other policy has hitherto been practicable. The non-panel men have been, and still are, in a minority, and consequently the first and most obvious of their duties was by standing aloof from its provisions to place on record the disfavour with which they regarded them. A minority's first duty is to make itself heard. Once it has done that, it can make use of the attention which is accorded to it. Aloofness was the first step towards having a policy, and this aloofness of non-panel medical men has been successful by bearing witness to the unsatisfactoriness of the medical arrangements, the public dislike of which is becoming increasingly obvious. The non-panel movement is a standing affirmation of the truth that contract practice on a large scale is a bad thing, and that a flat rate for all and sundry is medically impossible, partly because those who can and would prefer to pay are given an unsatisfactory medical attendance, and partly because the amount of work which contract practice on such a vast scale involves on the doctors has a bad effect both on private and contract work."

"Contract practice under the Act puts a premium on bad work?"

"Yes; take only a small instance. Two shillings is set down for drugs and appliances per head per annum. If the panel doctor keeps the cost of these down to eighteenpence he gets the other sixpence. This is really a sixpenny bribe to keep down the drug bill, and it means that a definite financial motive is officially offered to the doctor to consider profit and loss before the needs of his patients. More generally, for these contract patients the Act makes no provision of institutional treatment or consultations, operations or anæsthetics. The money which ought to be spent on these vital matters is frittered away on drugs. It is the fact that any medical man who makes a living mainly or wholly out of contract practice has to be inefficient from the amount of work he must undertake in order to make an adequate income."

"Is contract practice inherently bad?"

"On a large scale, certainly. But it is a necessity for the very poor, which is precisely the class of person so largely untouched by the Insurance Act. It cannot be too strongly emphasised that the medical profession would have accepted contract practice under the Act had such practice been restricted to those, such as the really poor, equitably entitled to receive that form of medical attendance.

### The Proper Basis of Medical Remuneration.

"Are you in favour of National Health Insurance as a principle?"

"My own opinion, publicly stated, has always been that to provide proper medical attendance for fourteen millions of people is an impossible problem, at least at present. Public opinion is not yet ripe for it. The cost of giving such a number adequate medical attendance is greater than the public is at present prepared to pay, and, apart from the cost, the public is not really yet educated up to the point of seeing the necessity for a national system of medical relief. But if such a course is really to be carried out efficiently, it is essential, in my opinion, that the basis of medical remuneration should be payment for work done, with the right of the medical committees to exclude those who, from their means and inclination (the two go together) have no need (and generally no desire) for contract practice. I myself favour voluntary insurance, but the difficulties are obvious. The good lives would probably want to stand out, because they could get better terms for themselves by doing so. Local associations of doctors would take voluntarily insured persons on favourable terms, and the Government scheme would be left to provide for the bad lives. The way in which these bad lives should be paid for is not by any flat rate, which is not actuarially practicable without throwing a very large share of their cost on the good lives (who naturally prefer the more favourable terms of companies or associations where bad lives are excluded), but by payment for work done. As the amount of this work is necessarily an unknown quantity, that is the only fair way of paying for it. Doctors are not insurance companies, and it is the curse of the present medical benefit arrangements that they force each panel doctor to become an insurance company in miniature. The result is, as we all know, the degradation of medicine from a science and art into a trade, and the dragooning of patients who are deprived of free choice of their medical attendant. The framers of the Act ignored the fact that medical attendance differs from any other kind of work. A medical visit or consultation is not (or should not be) an incident over in a moment, like buying a pound of tea over the counter. There is first a personal relation in it, and secondly a medical visit is a complex thing, requiring time and intimate knowledge as well as expert training. Diagnosis is not a single attempt at divination; it is the deduction from a complex series of observations in the light of experience and scientific training. Consequently, patients cannot pass through a surgery as customers pass through a shop. The Insurance arrangements have tried to make them do so, with disastrous results to all concerned."

"The by-products of the Act are bad?"

"They are certainly bad, and one great evil has been the degradation of medical ethics. For instance, I have lost a large number of insured persons from my practice because they weren't allowed to make their own arrangements with me unless I signed a paper which practically made me a panel doctor in respect of them. In no case did I receive, nor have I heard that any other doctor similarly placed has ever received, a notification from the doctor on whose list they were placed. I do not suggest that a panel doctor can be expected to find time to acknowledge the transference of patients to him. It is clear that he cannot; in other words, the custom of communicating with the previous medical attendant has disappeared, so far as panel practice is concerned. Now you cannot have such a state of things without not merely the ethical standard but the practical efficiency of medical practice suffering. Indeed, the protest against medical benefit is now coming loudly from the patients themselves.

### The Plight of the Panel Doctor.

"The rush of work is so great that not merely have diagnosis and treatment been scamped inevitably, but some panel men have gone so far as to decline certain cases, such as confinements, or to accept them only for inflated fees, on the ground, true enough, that they take up too much time. The result is that a patient of this class either has to pay at an enhanced rate for attendance which is guaranteed to her by the State in return for the taxation to which she is subjected, or else to rely on unskilled attendance. The position of the panel doctor is this. If he devotes to the confinement case the time and attention which it demands he is liable to receive a sharp reminder from the Insurance Committee that numbers of his other patients are being neglected. This pressure of work not only makes it out of the question for panel men to attempt to keep abreast of medical developments, but it also destroys the clinical experience which in the old days was often found a wonderful substitute for it. The great clinician, not necessarily the man with a fashionable practice, often used to adopt a good-humoured scepticism as to medical advances. But then his own specialty, which was clinical experience, was really something to be proud of and the result and reward of years of skill and patient work. To talk of clinical experience when patients are passed in and out of a surgery at the rate of a hundred or more a night is to exaggerate human powers of observation and to ignore the limitations of the clock; and, as for keeping abreast of the times, the picture of a panel practitioner sitting down to a quiet hour of study at the end of a long and busy day is one that excites a smile at its fantastic impossibility. It need hardly be said that it is unfair to attack the panel man individually, or to assume that in no case is he doing good work. There must also be, amongst fourteen millions of people, large numbers now receiving better attendance than they ever had before. But this does not affect the fact that panel practice as a whole is bad, and that its establishment marks a retrograde step from the standpoint of public and profession alike."

### How the Non-Panel Men are Justified.

"Do you think that the Act is breaking down?"

"Whether we look at the growing dissatisfaction of the public and the doctors—for the panel men are coming on evil times—or whether at the fear of bankruptcy which is spreading throughout the approved societies, its breakdown seems to be imminent. And this insolvency is one which non-panel men cannot fail to note with interest, for it confirms and strengthens the policy of aloofness which they have adopted from the first. It cannot now be pretended that political motives have influenced non-panel men, or that they were intent on 'smashing' the Act. Their support, it is now evident, could not have saved it, while its breakdown justifies their refusal to be entangled in its provisions. The late Dr. J. E. O'Sullivan, of Liverpool, was a strong supporter of the Government but an uncompromising opponent of the panel system, which he did his best to expose by organising the non-panel movement in that city. He was only a type of many who have never had any political bias in their dislike of the Insurance Act. A very good instance of the way in which the anomalies of the Act are alienating even those who began by being vociferous in its support may be seen in the change of view now adopted by many of the medical men who until recently were apparently well pleased with the panels they had assisted to set up, but are now calling loudly for a State medical service of a totally different character."

### Non-Panel Men on Insurance Committees.

"What, then, should be the non-panel policy?"

"While non-panel men have always thought it essential for them to avoid most scrupulously taking any paid work under the Act, some are still of opinion that it is good policy for non-panel men to accept such unpaid posts as those on Insurance and local Medical Committees. It is true non-panel members of these committees will always be in a minority and always be out-voted, yet it is urged by those who favour this permeation policy that it lets the non-panel men fully into what is going on, and as such is a negative, if not a positive, advantage. For this reason the National Medical Union left it to each local Non-Panel Association to frame its own rules as to whether or not it would permit its members to take these unpaid posts. The Medical Guild of Edinburgh has been unflinching in its attitude of allowing its members to take no service at all under the Act, and this seems to me the logical position to adopt, for I have never heard of any good resulting to the non-panel cause as the effect of the presence of non-panel men on insurance committees."

"The non-panel movement is growing?"

"Undoubtedly; and the National Medical Union will receive a great impetus as a result of its effectual protest in the matter of the proposed Statutory Medical Committee for London. Out of twenty-eight boroughs approached by the Metropolitan Branch of the British Medical Association sixteen have not nominated a single non-panel man to represent them, which is clearly the result of the circularisation of non-panel men by the National Medical Union. The National Medical Union pointed out that the proposed committee would not be legally constituted, as it would not be representative of the practitioners resident in the area, as required by the Act. Non-panel men were prompt to recognise this, and to follow the Union's advice not to allow themselves to be nominated for election. The Union is the only body which represents the non-panel cause, and it is the duty of every non-panel man to support it by becoming a member at once. The disgraceful shifts which have been adopted to bolster up the panel service can nowhere be better seen than over the squabble as to the spending of the surplus funds in the hands of the local committees. Any such funds were categorically promised by the Chancellor of the Exchequer to the insured persons to help them to pay their medical bills. But had this promise been kept the insured would have gone to non-panel men. To bolster up the panel, therefore, the insured had to be deprived of their due; and the one evident thing about the surplus funds is that panel practitioners, even if they should be legally (which is not as yet decided), are certainly not in equity, entitled to them. Finally, do not let panel men deceive themselves. As I said, they are coming on evil times. Any man who doubts this has only to see what has happened in Germany; here as there, moreover, the turn of the screw will be applied, slowly but surely. The process will be gradual to prevent a panic. Probably reductions of payment will be made by such degrees as from 7s. to 6s. 10d., and where is the panel man who, even if he sees that this is merely the beginning, will be firm and refuse at this price? Let the panel practitioners beware of the actuarial investigation into the financial condition of the approved societies, which may be expected to take place in 1915."

